# Giant cell tumor of tendon sheath in the hand: analysis of risk factors for recurrence in 50 cases

**DOI:** 10.1186/s12891-019-2866-8

**Published:** 2019-10-21

**Authors:** Hakan Ozben, Tamer Coskun

**Affiliations:** 1 Department of Orthopaedics and Traumatology, Hand Surgery Unit, School Of Medicine, Koc University, Davutpasa Street, No: 4 Zeytinburnu, 34010 Istanbul, Turkey; 2 Department of Orthopaedics and Traumatology, Hand Surgery Unit, Koc University Hospital, Istanbul, Turkey

**Keywords:** Giant cell tumor, Tendon sheath, Hand, Recurrence, Risk factors

## Abstract

**Background:**

Giant cell tumor of the tendon sheath is the most common form of giant cell tumors and is the second most common soft tissue tumor of the hand region after ganglion cyst. Magnetic resonance imaging is the diagnostic tool of choice for both diagnosis and treatment planning. The current standard treatment of choice is simple excision. The main concern about the treatment is related to the high recurrence rates. Besides incomplete excision, there is no consensus concerning the effect of other risk factors on recurrence. The literature lacks detailed reports on surgical excision of these tumors with a standardized surgical treatment and an appropriate patient follow up. The aim of this study was to investigate the recurrence rate and the associated recurrence risk factors for giant cell tumor of tendon sheath of the hand following a standardized treatment.

**Methods:**

The records of patients treated for giant cell tumor of tendon sheath of the hand treated by the same hand surgeon were evaluated retrospectively. The features obtained from preoperative magnetic resonance imaging, final physical examination, patients’ age and sex, anatomical site of the tumor, relationship of the tumor with bone, joint or neurovascular structures, bone invasion, recurrence after surgery and complications like skin necrosis, digital neuropathy or limitation in range of motion were documented. Chi-square test was used to compare categorical variables.

**Results:**

Fifty patient were included in the study. The average follow-up time was 84 months. Three recurrences (6%) were recorded. The only significant risk factor for the recurrence was tumor adjacency to the interphalangeal joints of the fingers other than thumb. No major or minor complications were encountered in the postoperative period.

**Conclusion:**

With adequate surgical exposure and meticulous dissection provided by the magnification loupes, we were able demonstrate one of the lowest recurrence rates in the literature. Well-designed studies combining the recurrence rates of several hand surgery centers implementing a standardized treatment are needed to better demonstrate the associated risk factors for recurrence.

## Background

Tenosynovial giant cell tumor, fibrous histocytoma of synovium, pigmented villonodular synovitis, localized nodular tenosynovitis, benign synovioma, fibrous xanthoma of the synovium are members of a family of lesions involving the joint synovia, tendon sheath and bursae; each one showing specific pathological features and being giant cell tumor of the tendon sheath (GCTTS) the most common form [1–5]. The pathogenesis of GCTTS is not clear [6]. Reactive or regenerative hyperplasia accompanied by an inflammatory process has been the most commonly accepted theory for GCTTS [6–8]. However, recent studies have demonstrated that most of these tumors exhibit chromosomal translocations involving chromosome 1p13 [9].

GCTTS is the second most common soft tissue tumor of the hand after ganglion cysts [1, 2, 4–8]. It occurs at any age with peak incidences in the third to fourth decades; women are mostly affected [1, 3, 4, 6, 7]. The tumor usually appears as a painless, slowly growing mass on the volar surface of the fingers [1, 3, 4, 6].

Diagnostic workup includes patient history and a detailed physical examination. Plain radiographs can be helpful since GCTTS may produce erosions in the cortical bone and may invade medullary space [3]. Magnetic resonance imaging (MRI) is the most useful diagnostic tool and is also required for surgical planning [3, 10]. MRI helps to classify GCTTS into type 1 and type 2 according to Al Qattan classification in which type I describes a single round or multilobulated tumor while type II describes two or more distinct, separated tumors [5, 6].

The current standard treatment of choice for GCTTS is simple excision [1, 4, 6, 8, 11, 12]. The main concern about the treatment is the high recurrence rates, ranging from 15 to 45% as reported by several studies [1–5, 7, 8, 11–13]. Incomplete excision is widely accepted as a definitive risk factor. Cortical destruction, location at the interphalangeal joint of the thumb and distal interphalangeal joints, presence of degenerative joint disease (DJD), type 2 tumors, tumors with increased mitotic activity, neurovascular dissection during removal and incomplete excision constitute risk factors favoring recurrence [1–15]. However, literature data are not sufficient to clearly identify which of the above listed pathologies may be more relevant in causing recurrence. Most of the mentioned studies contain series in which patients were not treated applying a standardized surgical protocol; also, the magnification power used during surgery was not stated. Furthermore, recurrence rates varied in different studies not presenting homogenous follow up periods or with a high number of lost patients.

In this study, we reviewed a series of 50 patients treated with a standard surgical set up by the same surgeon, in order to better characterize the factors associated to a high recurrence rate.

## Methods

Between 2005 and 2016, all patients who had been diagnosed histologically with GCTTS distal to the wrist joint, treated by the same surgeon and followed for at least 3 years, were retrieved from a prospectively enrolled database and analyzed retrospectively. Records of 50 patients were retrieved (34 female and 16 male) from the database. Each hand soft tissue tumor registry was created using a standardized format and inserted into the database. The standard registry format included the demography, a preoperative MRI of the involved lesion, detailed surgical notes and follow-up notes.

The data were completed by the report of the final physical examination assessing any tumoral formation with palpation, integrity of pulp sensation with response to light touch and measuring finger joint motions with a goniometer; anatomical site of the tumor, relationship of the tumor with the bone, joint or neurovascular structures, bone invasion, recurrence after surgery and complications like skin necrosis, digital neuropathy or limitation in the range of motion were also documented.

Al-Qattan classification was used to categorize the lesions as type I and type II according to MRI and intraoperative findings [5].

Tumor location was defined by the correspondence of the largest clinical appearance of tumor involvement to the anatomic landmarks of the hand such as the distal phalanx, distal interphalangeal (DIP) joint, middle phalanx, proximal interphalangeal (PIP) joint, proximal phalanx, metacarpophalangeal (MP) joint, metacarpal and carpometacarpal (CMC) joint. Thumb interphalangeal (IP) and MP joints were regarded as separate joints. Dorsal, volar or lateral localizations were also documented.

Tumors were also defined as being adjacent to periosteum, joint or neurovascular structure when MRI showed that lesions were in direct contact with these structures.

Bone invasion was preoperatively confirmed with MRI and supported by intraoperative findings.

All cases were operated using standard field 4.5x magnification loupes under tourniquet control. Tumors localized at the dorsal surface were removed using dorsal incisions. Midlateral incisions were used for laterally localized tumors. Tumors localized at the volar surface were approached with either lateral or volar incisions. Utmost care was given to remove the tumor in one piece with the capsule intact whenever possible and to protect digital nerves, arteries and tendons. En bloc excision were also performed for the tumors which invaded medullary space. Before the closure of the incision closure, the operation field was inspected for any satellite lesions. Standard postoperative management involved active finger motion at the end of the first week, suture removal at the end of second week and physiotherapist supervised rehabilitation until a full active range of motion was reached.

The study was performed within the guidelines of the hospital institutional review board and ethical committee approval was obtained (2019.252.IRB2.074).

### Statistical analysis

Statistical tests were performed with a commercially available software program (Statistical Package for the Social Science (SPSS) 20.0 for Windows, Chicago, IL, USA). Continuous variables were presented as mean ± standard deviation while categorical variables were expressed as numbers. Chi-square test was used to compare categorical variables. *P* < 0.05 was considered statistically significant.

## Results

**Fig. 1:**
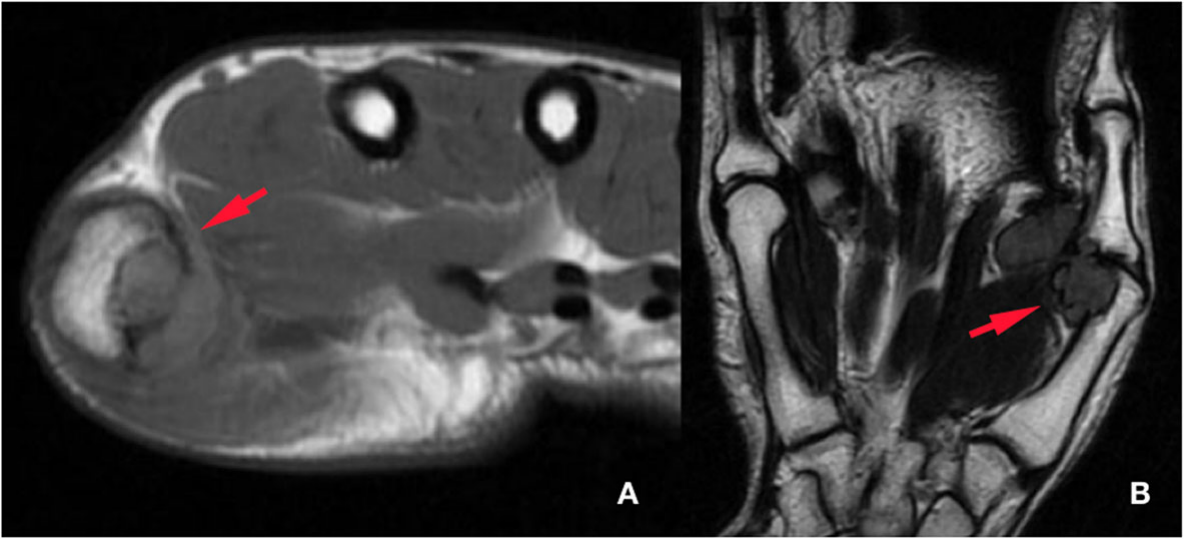
Axial (**a**) and coronal (**b**) MRI shows that GCTTS of thumb ray has penetrated into metacarpal head and metacarpophalangeal joint

**Fig. 2:**
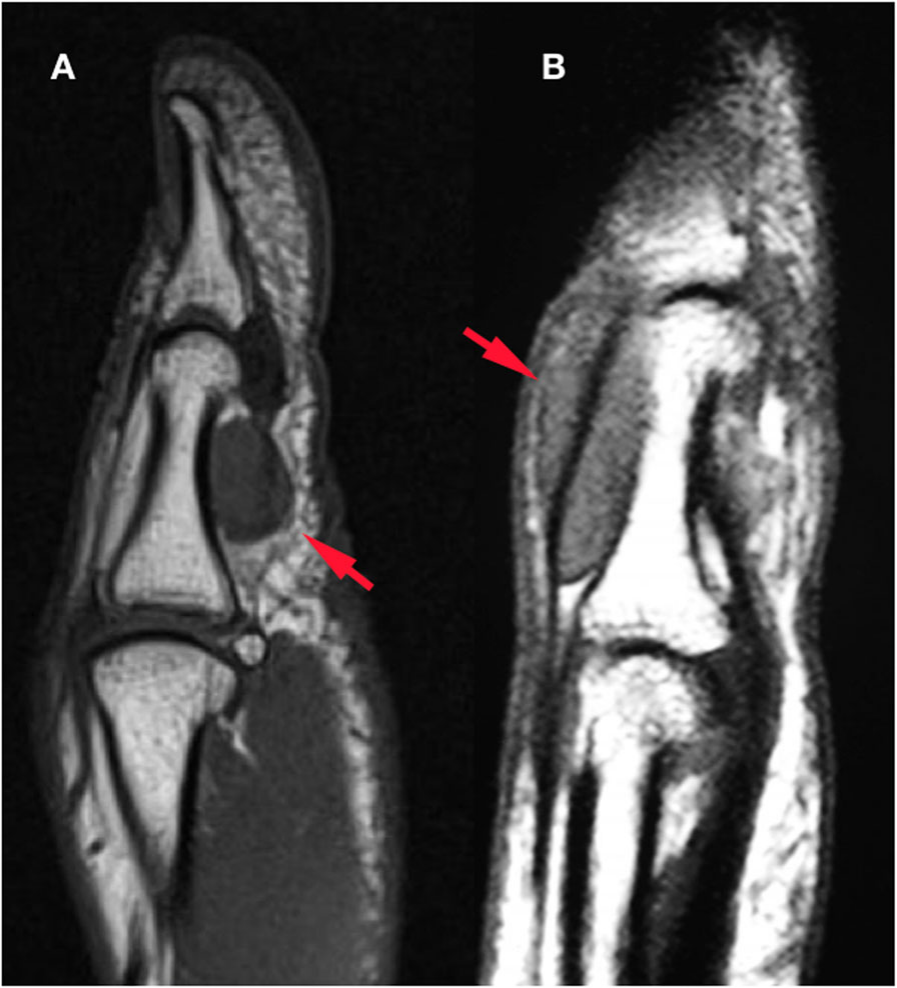
MRI clearly shows periosteal contact in GCTTS that was localized volarly (**a**) and dorsally (**b**)

**Fig. 3:**
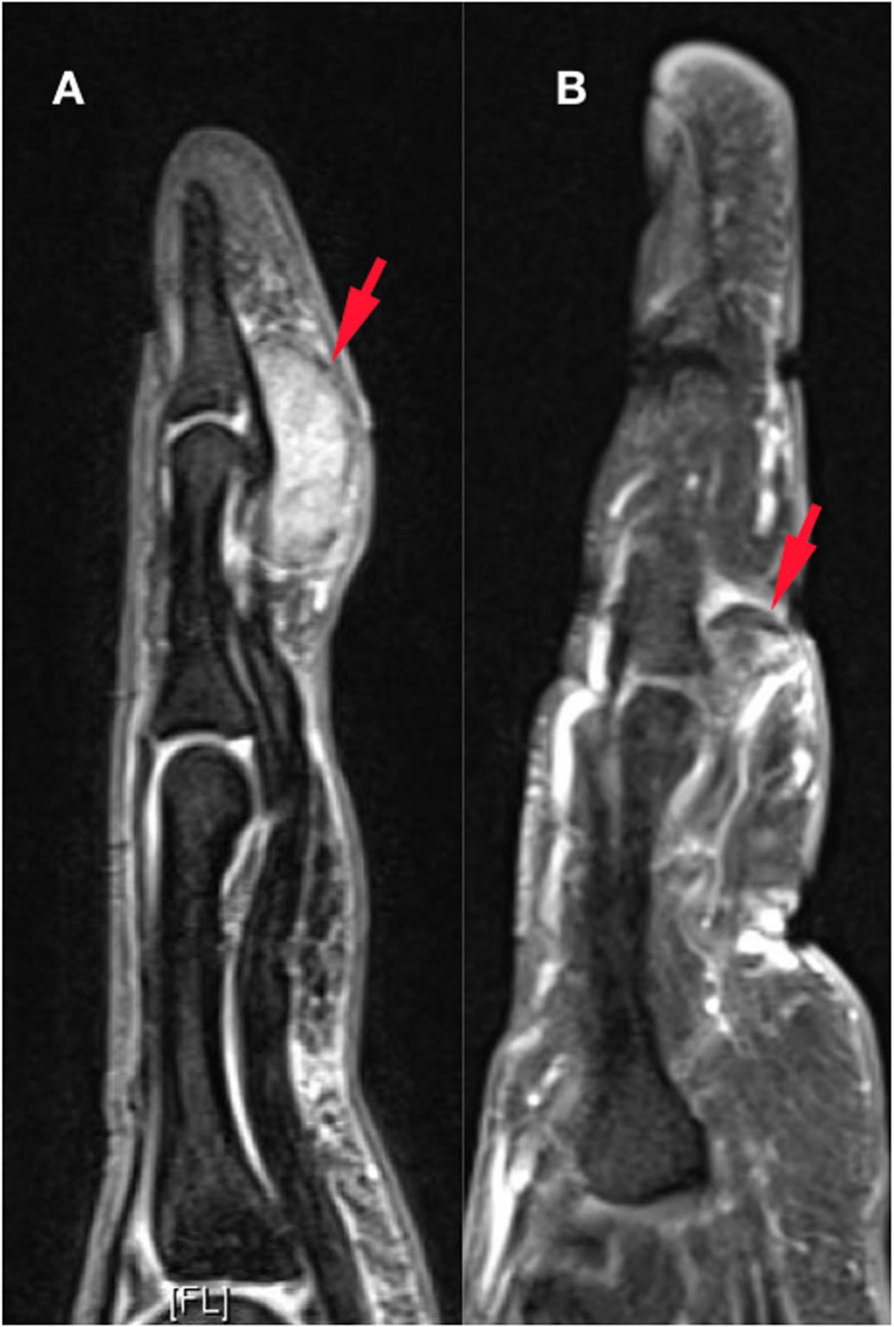
MRI may reveal GCTTS proximity to DIP (**a**) and PIP (**b**) joints

**Fig. 4:**
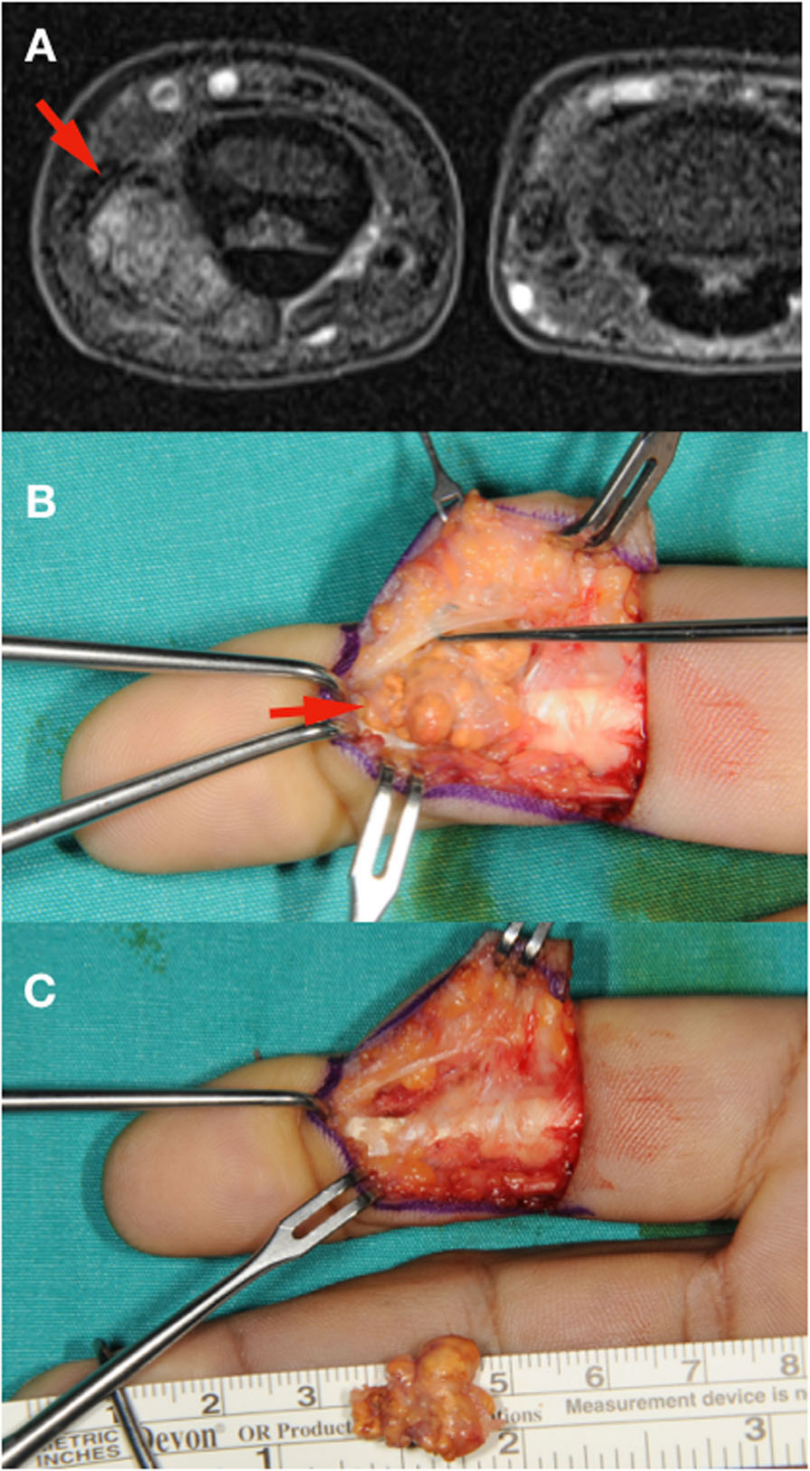
Neurovascular bundle involvement is suspected with a GCTTS localized to the lateral aspect of the flexor tendon (**a**). The tumor was dissected carefully (**b**) and the integrity neurovascular structures was preserved (**c**)

**Fig. 5:**
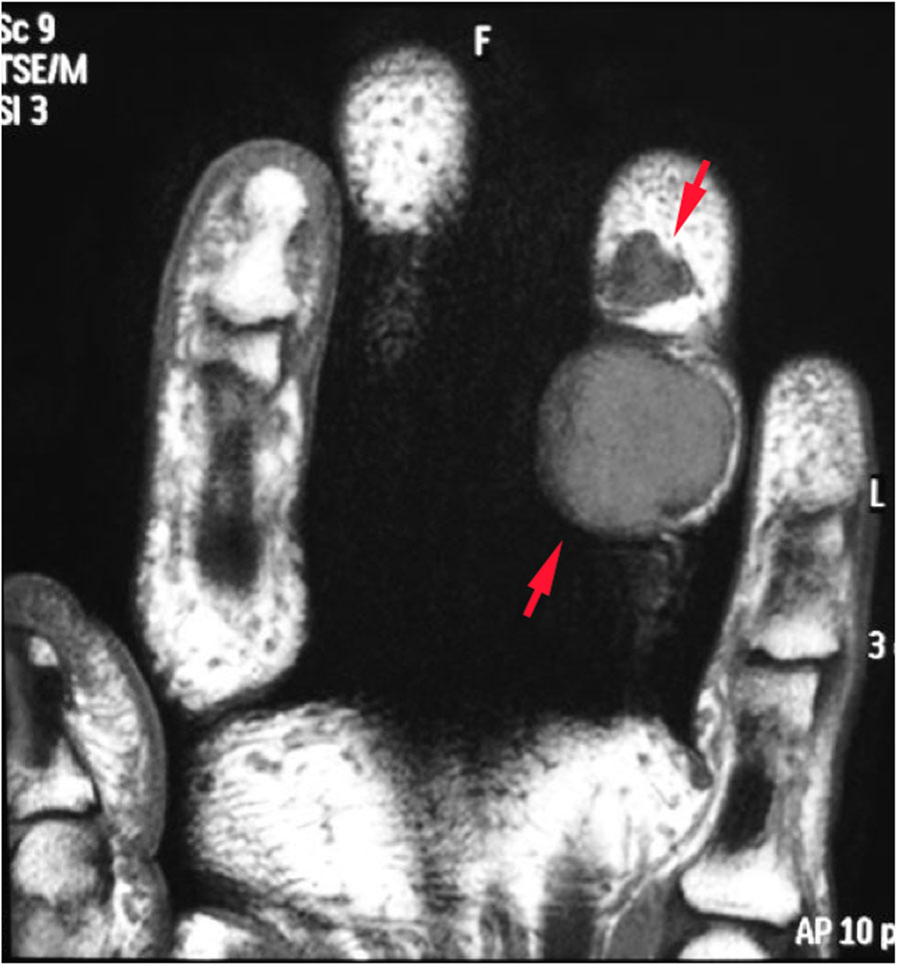
MRI can identify Al Qattan type 2 tumors where 2 or more distinct tumoral lobes exist

All 50 patients were included in the study and no patient was lost during the follow-up. The mean age of the patients at the time of operation was 44.4 ± 13.3 years (range: 18 years - 69 years). The average follow-up time was 84.3 ± 43.2 months (range: 38 months – 173 months). Anatomical localization and specific distribution of the tumors are shown in Table 1. In one patient, tumor penetrated into the thumb MP joint and thumb metacarpal head (Fig. 1). In another two cases, the tumor destroyed bone cortex and invaded the medullary space. Tumors were in direct contact with periosteum in 14 patients (Fig. 2). In ten patients, tumors were adjacent to DIP and PIP joints of fingers (Fig. 3). Tumor localization necessitated digital nerve and artery dissection in 34 patients (Fig. 4). According to Al Qattan classification, 34 tumors were type 1 while 16 tumors were type 2 (Fig. 5). Pathology reports demonstrated no tumoral continuity in surgical margins in any of the patients.

**Table 1:** Anatomical distribution of tumors

	Distal Phalanx	DIP	Middle Phalanx	PIP	Proximal Phalanx	Thumb Distal Phalanx	Thumb IP	Thumb Proximal Phalanx	MP	Thumb MP	Metacarpal
Volar	3	5	3	1	6	0	3	4	4	3	0
Lateral	0	3	2	3	1	0	0	1	0	0	0
Dorsal	0	4	1	2	0	0	0	0	0	0	1
Total	3	12	6	6	7	0	3	5	4	3	1

Recurrences were seen in 3 patients (overall recurrence rate: 6%). The first recurrence case was a 33-year old female whose tumor was localized to the lateral side of the DIP joint of the little finger. It was a type 2 tumor and neurovascular dissection was carried out to protect digital nerves. The recurrence was seen in the postoperative 4th year and was treated with re-excision. The second recurrence case was a 50-year old female patient whose tumor was localized to the lateral side of the DIP joint of the index finger. It was a type 1 tumor and neurovascular dissection was carried out to protect digital nerves. The recurrence was seen at the postoperative 18th month and was treated with re-excision. The third case of recurrence was a 68-year old female patient whose tumor was localized to the lateral side of the PIP joint of the index finger. It was a type 2 tumor and tumor removal did not require neurovascular dissection. The recurrence was seen at the postoperative 20th month and the patient refused re-excision and is being followed.

Table 2 demonstrates that, regarding tumor proximity to the osteoarticular structures, recurrences was significantly higher in tumors adjacent to interphalangeal joints. No recurrence was seen in tumors adjacent to periosteum or in tumors with bone invasion as well as in tumors with no contact with bone or joints.

**Table 2:** Recurrence rate regarding proximity to osteoarticular structures

	Bone invasion (*n* = 3)	Periosteum adjacency (*n* = 14)	PIP/DIP joint adjacency (*n* = 10)	No direct contact (*n* = 23)	*P*
Recurrence (+)	0	0	3	0	0.005
Recurrence (−)	3	14	7	23	

A significant difference between groups was considered for *p* < 0.05

Tables 3 and 4 shows recurrence rates in cases regarding to neurovascular dissection necessity and Al Qattan type respectively. Neurovascular dissection or Al Qattan type 2 did not significantly increase recurrence rate.

**Table 3:** Recurrence rate regarding neurovascular dissection

	Neurovascular dissection (+) (*n* = 34)	Neurovascular dissection (−) (*n* = 16)	*P*
Recurrence (+)	2	1	1.0
Recurrence (−)	32	15	

A significant difference between groups was considered for *p* < 0.05

**Table 4:** Recurrence rate regarding Al Qattan classification

	Type 1 (*n* = 34)	Type 2 (*n* = 16)	*P*
Recurrence (+)	1	2	1.0
Recurrence (−)	33	14	

A significant difference between groups was considered for *p* < 0.05

Patients with tumors adjacent to interphalangeal joints, neurovascular dissection or Al Qattan type 2 tumor were not exposed to a statistically higher risk of recurrence (Tables 5 and 6).

**Table 5:** Recurrence rate regarding neurovascular dissection among tumors adjacent to IP joints

	Adjacent to Joint, neurovascular dissection (+) (*n* = 6)	Adjacent to Joint, neurovascular dissection (−) (*n* = 4)	*P*
Recurrence (+)	2	1	1.0
Recurrence (−)	4	3	

A significant difference between groups was considered for *p* < 0.05

**Table 6:** Recurrence rate regarding Al Qattan classification among tumors adjacent to IP joints

	Adjacent to Joint, type 1 (*n* = 4)	Adjacent to Joint, type 2 (*n* = 6)	*P*
Recurrence (+)	1	2	1.0
Recurrence (−)	3	4	

A significant difference between groups was considered for *p* < 0.05

At the last follow up control, every patient showed the same range of motion as compared to the contralateral hand. Sensorial examination was normal. All patients reported wound healing and suture removal within 2 to 3 weeks after surgery without any skin necrosis or local infection.

## Discussion

Notwithstanding the high number of published papers on hand GCTTS, the treatment represents still a challenge for the hand surgeon. Since the tumor may penetrate into joints and bone cortices, extend into tendon sheaths and enclose neurovascular structures, establishing the correct balance between a complete and aggressive tumor removal along with the preservation of vital tissues poses major difficulties. In this study, we analyzed the recurrence rate in our patient series and investigated the correlation between recurrence and known risk factors. We found the overall recurrence rate as 6% and joint capsule adjacency as the only significant risk factor.

The recurrence rate after surgical excision has been reported as high as 15–45% [1–5, 7, 8, 12, 13]. Several risk factors have been shown to be associated with higher recurrence risk. Table 7 summarizes the literature data regarding the recurrence rate in various series.

**Table 7:** Rates of recurrences in the literature

Paper	Total number of cases^b^	Cases with known risk factor	Recurrence rate in high risk cases	Overall recurrence rate (%)	Follow up (months)
Wright (1951) [16]	54	^a^	^a^	44	1–120
Sherry and Anderson (1955) [17]	12	^a^	^a^	25	12–72
Jones (1969) [18]	72	53 joint involvement	^a^	22	1–120
Fyfe and MacFarlane (1980) [19]	51	30 joint involvement	^a^	38	6–240
Rao and Vigorita (1984) [20]	17	^a^	^a^	29	3–108
Moore et al. (1984) [21]	115	53 joint involvement, 10 bone involvement	^a^	9	1–324
Grover et al. (1998) [22]	52	7 bone involvement, 13 tumor was Al Qattan type 2	57% with bone involvement; 30% with Al Qattan type 2	15	7–174
Looi et al. (1999) [23]	53	21 bone involvement	^a^	7	12–60
Reilly et al. (1999) [8]	70	10 bone involvement, 30 joint involvement	50% with bone involvement; 42% with joint involvement,	27	7–138
Kotwal et al. (2000) [13]	48	^a^	^a^	4	24–132
Al-Qattan (2001) [5]	43	13 tumor was Al Qattan type 2	38% with Al Qattan type 2	11	24–72
Ozalp et al. (2004) [24]	134	^a^	^a^	16	6–132
Kigawa et al. (2004) [12]	30	3	^a^	13	12–126
Lowyck and De Smet (2006) [25]	43	27 joint involvement, 8 bone involvement	0% with bone involvement; 14% with joint involvement	16	15–136
Darwish (2008) [2]	52	^a^	^a^	24	36–120
Williams et al. (2010) [10]	213	10 bone involvement, 40 joint involvement, 23 neurovascular involvement	10% with bone involvement; 30% with joint involvement, 22% with neurovascular involvement	13	36-^a^
Di Grazia et al. (2013) [4]	64	7 neurovascular involvement	42% with neurovascular involvement	4.7	2–153
Koutserimpas et al. (2018) [6]	36	9 tumor was Al Qattan type 2	^a^	11	^a^

^a^Not reported

^b^Number of cases included in the study

Specific anatomical sites such as interphalangeal joints of the fingers and thumb localization have been shown to be associated with higher recurrences [2, 4, 7, 8]. Reilly et al. [8] reported that recurrence was higher with tumors localized at DIP joints and thumb IP joint (79%) and with dorsally localized tumors (34%). In the series of Williams et al. [10], there were 3 recurrences in 34 tumors localized to the thumb. However, Fotiadis et al. [5] reported that a specific finger or phalanx was not associated with a higher risk for recurrence. Although a statistical analysis was not possible due to the small number of recurrences in our series; recurrences were seen only at PIP and DIP joints. On the contrary, no recurrence was seen after the excision of 11 tumors localized to the thumb ray and in dorsally localized tumors.

Tumors with close relationship with bone, joint and neurovascular structures have also been shown to recur more frequently [2, 4, 5, 7, 8]. Al Qattan et al. [5] mentioned that intra-osseous invasion of GCTTS may carry a higher risk for recurrence. Cortical contact and bone erosions and invasion were shown to be risk factors for recurrence and Reilly et al. [8] reported that recurrence was seen in 5 patients out of 8 with bone erosions. In contrast, our results showed that bone invasion and cortical proximity did not correlate with recurrence. Our findings support the reports stating that bone involvement was due to simple erosion or invasion by the pressure effect of the tumor [8, 12, 25].

A few studies investigated the involvement of certain structures on recurrence. Williams et al. [10] showed that recurrence rates reached 32% when the flexor, extensor tendons and joint capsule were involved. Reilly et al. [8] reported a recurrence rate of 58% with tumors that were in contact with PIP or DIP joints. Kitagawa et al. [12] stated that tumor proximity to neurovascular structures rendered complete tumor excision difficult and was associated with a higher recurrence rate. Di Grazie et al. [4] reported 3 recurrences in 7 patients with neurovascular bundle involvement. Our results were partly in accordance with the literature: Only joint capsule adjacency was shown to be a statistically significant risk factor for recurrence, whereas no correlation was found for neurovascular bundle involvements. In our study, neurovascular dissection did not increase recurrence risk in tumors adjacent to joints. It is also important to mention that, in all recurrent cases, the primary tumor was localized to either side of the fingers with no extension into tendon sheaths, which means that no recurrences were seen in cases where tumors were dissected from flexor or extensor tendons.

Al Qattan et al. [5] have reported that type 2 tumors are associated with higher recurrence rates. This theory is supported by other papers as well [4, 7]. Our results, in contrast, did not support this statement, type 2 tumors not showing an increased recurrence rate. Moreover, regarding tumors in contact with the joints, being a type 2 tumor did not bring any additional risk for recurrence.

We can confidently state that the single most important factor to prevent recurrence is the complete surgical excision [2, 5, 7]. The importance of magnification during surgical excision to achieve complete tumor removal has been emphasized by several studies [4, 6, 11, 15]. Williams et al., summarized the overall recurrence rates in several studies ranging between 7 and 44% [10]. In our study, the overall recurrence rate was 6%. The explanation for the low recurrence rate in our study is the achievement of complete tumor excision by an experienced hand surgeon using 4.5x magnifying loupes, magnification being of utmost importance to obtain a thorough tumor dissection from periosteum, joints, neurovascular and tendinous structures and avoiding any significant complication.

We are convinced that the highly positive results of our study lie in the adoption of a standardized surgical setup applied by an experienced surgeon. The patients required minimum follow up and no patients were lost during the follow-up. A follow-up of 3 years and more may be considered sufficient to rule out future recurrences.

Our study has some limitations. Firstly, the recurrence number was too small and prevented statistically certain assertions. However, achieving a small recurrence rate is one of the goal of GCTTS treatment. Secondly, not all patients had standard radiographies of the involved sites. Some patients were referred from other medical facilities with hand MRIs only. Since MRI clearly showed details of bone involvement and tumor extension, we thought that standard x-rays would not give additional information for surgical planning. Furthermore, several papers reported that pressure erosions and arthritic changes, as documented by x-rays, were not associated with a higher recurrence rate [25]. Moreover, one of the recurrences in our series was seen at the age of 37 which could be interpreted as a young age to expect DJD. We did not apply postoperative radiotherapy since it is not widely accepted as an adjuvant therapeutic application [2, 4, 7, 11]. Kotwal et al. [13] reported no recurrence in 14 patients with postoperative radiotherapy. However, other studies reported 2.3–75% recurrences with postoperative radiotherapy [7, 11, 18]. Although treatment of all patients by the same experienced surgeon might be seen as a study limitation, we tried to eliminate the effect of incomplete excision and inadequate surgical competency on recurrence rates. A final observation concerns the fact that as all procedures were performed with the use of X 4.5 magnifying loupes, we could not compare the effect of the use of loupes with lower magnification.

## Conclusion

Complete surgical excision remains the gold standard treatment for GCTSS. Recurrence is the single most important issue that preoccupies both the patient and the treating surgeon. Besides incomplete excision and joint involvement, there is no consensus on the effect and contribution of previously identified risk factors for recurrence. Thanks to an adequate surgical exposure and meticulous dissection provided by the magnification loupes, we had one of the lowest recurrence rate reported in the literature. Well-designed studies combining the recurrence rate of several hand surgery centers with standardized treatments are needed to better demonstrate the associated risk factors for recurrence.

## Data Availability

The datasets used and/or analyzed during the current study are available from the corresponding author on reasonable request.

## Abbreviations

CMC: Carpometacarpal
DIP: Distal interphalangeal
DJD: Degenerative joint disease
GCTTS: Giant cell tumor of the tendon sheath
IP: Interphalangeal
MP: Metacarpophalangeal
MRI: Magnetic resonance imaging
PIP: Proximal interphalangeal
